# MicroRNA367 negatively regulates the inflammatory response of microglia by targeting IRAK4 in intracerebral hemorrhage

**DOI:** 10.1186/s12974-015-0424-3

**Published:** 2015-11-09

**Authors:** Bangqing Yuan, Hanchao Shen, Li Lin, Tonggang Su, Lina Zhong, Zhao Yang

**Affiliations:** Department of Neurology, Yongchuan Hospital, Chongqing Medical University, Chongqing, 402160 China; Department of Neurosurgery, The 476th Hospital of PLA, Fuzhou, Fujian 350025 China

**Keywords:** microRNA367, Inflammation, Microglia, IRAK4, ICH

## Abstract

**Background:**

Intracerebral hemorrhage (ICH) induces microglial activation and the release of inflammatory cytokines, leading to inflammation in the brain. IRAK4, an essential component of the MyD88-dependent pathway, activates subsets of divergent signaling pathways in inflammation.

**Methods:**

In the experiment, microglia were stimulated with erythrocyte lysates, and then miR-367, IRAK4, NF-ĸB activation and downstream proinflammatory mediator production were analyzed. In addition, inflammation, brain edema, and neurological functions in ICH mice were also assessed.

**Results:**

Here, we report that ICH downregulated miR-367 expression but upregulated IRAK4 expression in primary microglia. We also demonstrate that miR-367 suppressed IRAK4 expression by directly binding its 3′-untranslated region. MiR-367 inhibited NF-ĸB activation and downstream proinflammatory mediator production. Knocking down IRAK4 in microglia significantly decreased the IRAK4 expression and inhibited the NF-ĸB activation and the downstream production of proinflammatory mediators. In addition, our results indicate that miR-367 could inhibit expression of proinflammatory cytokines, reduce brain edema, and improve neurological functions in ICH mice.

**Conclusions:**

In conclusion, our study demonstrates that miR-367/IRAK4 pathway plays an important role in microglial activation and neuroinflammation in ICH. Our finding also suggests that miR-367 might represent a potential therapeutic target for ICH.

## Background

Intracerebral hemorrhage (ICH) is a particularly devastating form of stroke with high mortality and morbidity [1–3]. Patients with ICH have poor prognosis, 80 % of those survivors beyond the acute phase may suffer prolonged neurological deficits and brain atrophies [4–6]. Brain damage after ICH is caused not only by the mass effect of hematoma but also by the secondary pathological processes. ICH causes secondary injury through various pathways, including the initiation of an acute inflammatory response, local release of reactive oxygen species (ROS), and perihematomal edema [7–9].

The TLR/IL-1R superfamily mediates the innate immune response mainly by upregulating the expression of inflammatory genes in multiple target cells [10–12]. A member of the IL-1 receptor (IL-1R)-associated kinase (IRAK) family, IRAK4, has been shown to play an essential role in Toll-like receptor (TLR)-mediated signaling [13–15]. IRAK4 kinase inactive knockin mice have been shown to be completely resistant to LPS- and CpG-induced shock, due to impaired TLR-mediated induction of proinflammatory cytokines and chemokines.

MicroRNAs (miRNAs) are key players in regulating inflammatory response. Some miRNAs have anti-inflammatory functions and serve as negative feedback regulator of inflammation [16–18]. Inflammatory stimuli, such as Toll-like receptor, interleukin (IL)-1β, or IL-13, can stimulate the upregulation of miR-147, miR-146a, or miR-21, respectively. These miRNAs either reduce inflammatory factor expression or suppress inflammatory response [17–19].

Previously, we used a miRNA microarray to detect the expression profiles of cellular miRNAs, and our unpublished observations found that the expression level of miR-367 was significantly downregulated in microglia treated with erythrocyte lysates. To further explore the potential target proteins of miR-367, we utilized the bioinformatic tool, TargetScan, and found that IRAK4 was the main target. In the present study, we aimed to investigate whether miR-367 was involved in the regulation of inflammatory responses via IRAK4 in ICH. Our present data identified that miR-367 was a crucial regulator of TLRs downstream NF-κB signaling by direct targeting IRAK4. These results suggested that miR-367 was a novel inflammatory regulator in ICH.

## Methods

### Microglia culture

Primary hippocampal microglia was isolated from glial cultures prepared from newborn (less than 24 h old) Sprague-Dawley (SD) rats (Laboratory Animal Center, Chongqing Medical University, Chongqing, China). Glial cells were cultured in 75 cm^2^ flasks for 14 days in DMEM/F12 (Gibco BRL, Grand Island, NY, USA) supplemented with 10 % FCS (Hyclone, Logan, UT, USA), 100 U/ml penicillin and 100 mg/ml streptomycin. Microglia was isolated from primary mixed glial cell cultures on day 10 by shaking the flasks overnight at 300 rpm on a rotary shaker at 37 °C. The purity of the microglial cultures was assessed as over 90 %, using a CD11b antibody (Santa Cruz, USA). Cells were cultured for 2 days before treatment. The Chongqing Medical Experimental Animal Care Committee approved the protocol for this study, and all animal experiments were conducted in accordance with the National Institutes of Health Guidelines for the Care and Use of Laboratory Animals.

### Preparation of erythrocyte lysates

Spleens were removed from SD rats. Single-cell suspensions of splenocytes were prepared using stainless steel mesh screens. And then, 1 × 10^5^ splenocytes were incubated with 1 ml red blood cell lysing solution for 20 min, and centrifuged at 2000 rpm for 10 min. The supernatants were utilized as **e**rythrocyte lysates.

### Cell treatment

Microglia (1 × 10^5^) was stimulated with 10 μl PBS or erythrocyte lysates for 48 h. After then, the supernatants were removed and further analyzed for cytokine production with ELISA.

### Real-time PCR

Total RNA was extracted from the microglia using TRIzol reagent (Invitrogen, San Diego, CA, USA) in accordance with the manufacturer’s instructions. Semi-quantitative real-time PCR, using SYBR Green I, was conducted to compare the relative expression levels of specific mRNAs. The sequences of primers used were shown as following: IRAK-4, 5′-GTCATGACCAGC CGAA TCGTG-3′ (forward), 5′-CAG ACA CT GGTCAGCAGCAGA-3′ (reverse); IL-6, 5′-AGCATACA GTTT GT GG ACATT-3′ (forward), 5′-CAACATTCATATTGCCAGTTCT -3′(reverse); IL-1β, 5′-CAGGCAACCACTTACCTATTTA-3′(forward),5′-CCATA CACAC GGACAACAACTAGAT-3′(reverse); TNF-α, 5′-C GAG TG AC AAGCCT GTAGC -3′(forward); 5′-TACTTGG GCAGAT TGACCTC A -3′(reverse); GADPH, 5′-CAT GGTCTACATGTTCCAGT-3′(forward); 5′-GGCTAAGCAGTTGGTGGT GC -3′ (reverse). TaqMan miRNA assays (Applied Biosystems Inc., Carlsbad, CA, USA) were used to quantify mature miRNA expression levels, in accordance with the manufacturer’s protocol. For each of the selected miRNAs, real-time PCR measurements were performed to obtain a mean CT value for each sample. The CT values of the different samples were compared using the 2-^ΔΔ^CT method, and U6 expression levels were used as an internal reference as previous methods [20]. All PCR experiments were done in triplicate on an ABI system in 384-well plates using the TaqMan Universal Master Mix II with the UNG kit. PCR primers were obtained from Applied Biosystems. Each run included water blanks and genomic DNA as negative controls. Each run consisted of 35 cycles with miRNA levels in samples without amplification signal considered zero.

### Western blotting analysis

Briefly, total protein was extracted from cultured cells and quantified using a commercial bicinchoninic acid (BCA) kit (BCA Protein Assay Kit; Pierce Biotechnology Inc., Rockford, IL, USA) with BSA as the standard. Equal amounts of protein from different cells were separated by 10 % SDS-PAGE and transferred to a nitrocellulose membrane (Bio-Rad, Hercules, CA, USA). The membrane was blocked with 5 % non-fat milk, and incubated with primary antibodies (IRAK4, NF-κB p65; Santa Cruz, USA). The blots were then incubated for 2 h at room temperature with horseradish peroxidase-conjugated secondary antibodies in blocking buffer. Target proteins were detected using an enhanced chemiluminescence kit (Amersham Pharmacia Biotech, Uppsala, Sweden).

### Enzyme-linked immunosorbent assay

Supernatants were measured by ELISA as specified by the manufacturer. Microglia plated in 24-well plates was cultured with anaerobic for 6 h. The culture supernatants were aspirated and stored at −70 °C until assayed by ELISA. The concentrations of IL-6, IL-1β, and TNF-α in the stored media were measured using a sandwich ELISA kit (R&D Systems).

### Oligonucleotide transfection

The control RNA duplexes for the miRNA mimics and the small interfering RNAs (siRNAs) were not homologous to any mouse gene sequences. The siRNAs were synthesized by targeting mouse IRAK4 transcripts. Oligonucleotide transfection was performed with commercial reagents (Lipofectamine 2000; Invitrogen Corp.). Each transfection used 50 nmol/l of RNA duplexes.

### Vector construction and luciferase reporter assays

To generate the miR-367 expression vector, the miR-367 gene was amplified from mouse genomic DNA and cloned into the pcDNA3.0 vector (Invitrogen Corp.). The luciferase complexes were constructed by ligating oligonucleotides containing the wild-type ( GUGCAAU) or mutated putative target site (ACAUGGC) of the mouse IRAK4 3′-untranslated region (UTR) into the multi-cloning site of the p-MIR luciferase reporter vector (Ambion Inc., Austin, TX, USA). BV-2 cells were cotransfected with 80 ng of the luciferase reporter plasmid, 40 ng of the pRL-TKRenilla-luciferase plasmid (Promega Corp., Madison, WI, USA), and the indicated RNAs (final concentration 20 nmol/l). At 24 h, after the transfection, the firefly and Renilla luciferase activities were measured (Dual-Luciferase Reporter Assay; Promega Corp.). Each transfection was repeated twice in triplicate.

### ICH model

Briefly, mice were anesthetized with an intraperitoneal injection of 400 mg/kg chloral hydrate and fixed on a mouse stereotaxic frame (Stoelting). A 20 μl volume of autologous nonanticoagulated blood was collected from the tail vein of the mouse and then injected into the caudate nucleus at 2 μl/min under stereotactic guidance at the following coordinates relative to bregma: 0.8 mm anterior, 2 mm left lateral, and 3.5 mm deep during a period of 10 min The needle was held in place for 10 min after injection, and the microsyringe was pulled out after the blood had coagulated. The craniotomy was then sealed with bone wax, and the scalp was closed with sutures. Body temperature was maintained at 37 °C throughout the procedure, and the mice were given free access to food and water after they woke up. The mice that died because of anesthesia were excluded.

### Intracerebroventricular injection

The in vivo transfection was performed according to the method described as follows: the stereotaxic coordinates were 0.5 mm posterior and 1.0 mm lateral to bregma and 2.5–3.0 mm ventral to the surface of the skull. The miR-367 mimics or miR-367 control (2 μg/2 μl) were added to 1.25 μl of Entranster™ in vivo transfection reagent. The solution was mixed gently, left for 15 min and then injected intracerebroventricularly (i.c.v.) using a micro syringe (Hamilton, NV, USA) under the guidance of the stereotaxic instrument (RWD Life Science).

### Evaluation of neurological scores

The neurological scores were determined by neurological severity scores, a composite of motor, sensory, reflex, and balance tests according to the reference [21]. And the timing of performing behavioral experiments was 48 h after blood infusion. Neurological function was graded on a scale of 1 to 18; a score of 1 point is awarded for the inability to perform the test or for the lack of a tested reflex. The higher the score, the more severe the injury (normal score 2–3, maximal deficit score 18).

### Brain water content measurement

Brain water content was measured in mouse cerebral tissues after ICH. Briefly, mice were randomly sampled from each group and anesthetized by intraperitoneal injection with chloral hydrate (*n* = 5). Next, the cerebral tissues were removed, and the surface water on the cerebral tissues was blotted with filter paper. Brain samples were immediately weighed on an electric analytic balance to obtain the wet weight and then dried at 100 °C for 24 h to obtain the dry weight. Brain water content was calculated using the following formula: brain water content (%) = (wet weight − dry weight)/wet weight × 100 %.

### Statistical analysis

The statistical significance of differential findings between experimental groups and controls were determined by ANOVA and post-hoc analysis, and considered significant if *P* < 0.05.

## Results

### Erythrocyte lysates upregulated IRAK4 expression but downregulated miR-367 expression in microglia

**Fig. 1:**
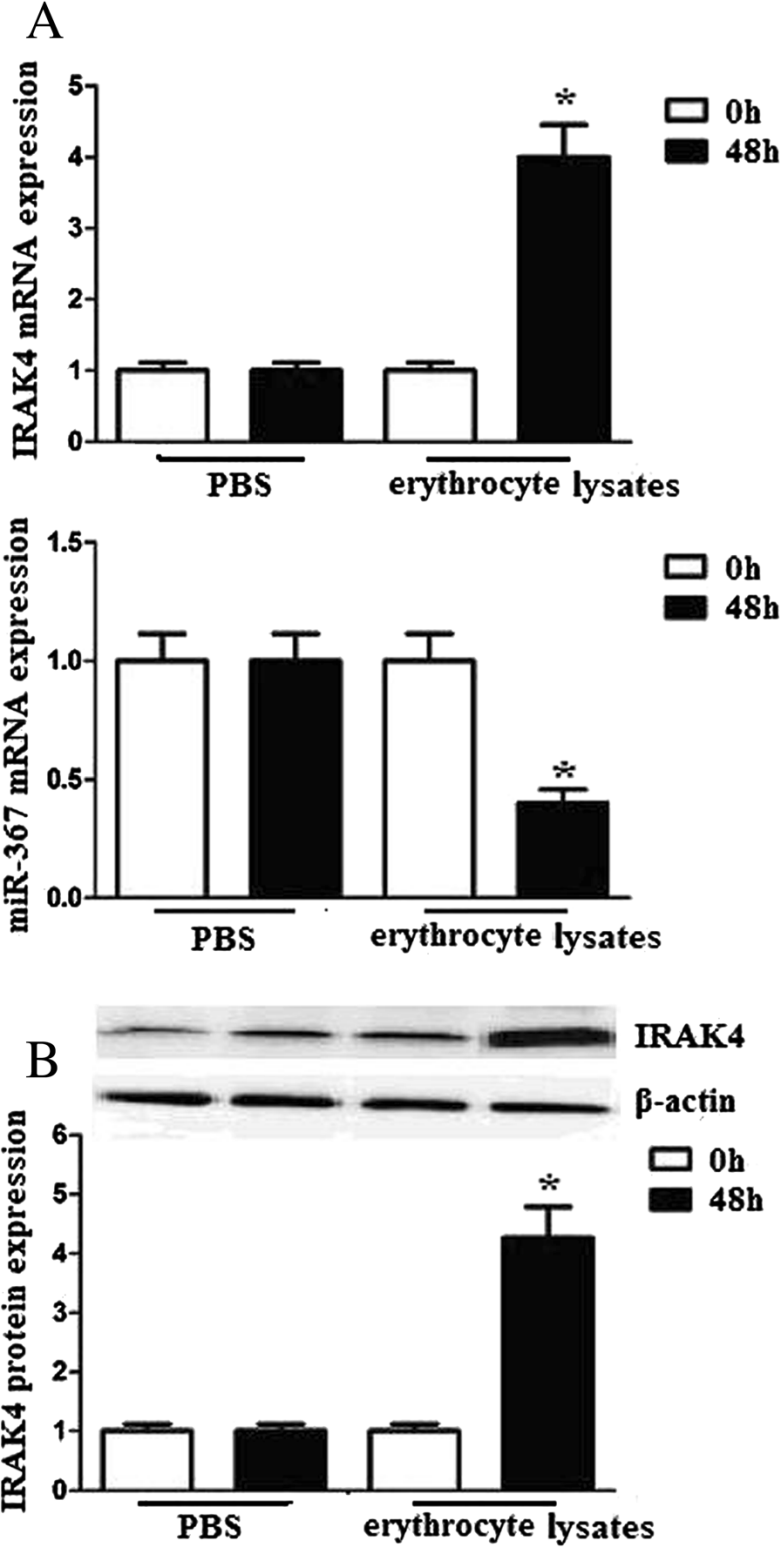
Erythrocyte lysates upregulated IRAK4 expression but downregulated miR-367 expression in microglia. Microglia (1 × 10^5^) was stimulated with 10 μl PBS or erythrocyte lysates for 48 h. **a** IRAK4 and miR-367 mRNA expression levels were evaluated by quantitative RT-PCR. **b** IRAK4 protein expression levels were evaluated by western blots. Experiments performed in triplicate showed consistent results. Data are presented as the mean ± SD of three independent experiments. *P < 0.05

We measured IRAK4 mRNA and protein expression of microglia 48 h after erythrocyte lysates or PBS treatment. We found that IRAK4 mRNA and protein expression increased after erythrocyte lysates treatment. On the contrary, miR-367 expression significantly decreased after erythrocyte lysates treatment (Fig. 1). These results demonstrated that miR-367 and IRAK4 expression of microglia have an inverse correlation after erythrocyte lysates treatment.

### miR-367 regulates IRAK4 expression in microglia

**Fig. 2:**
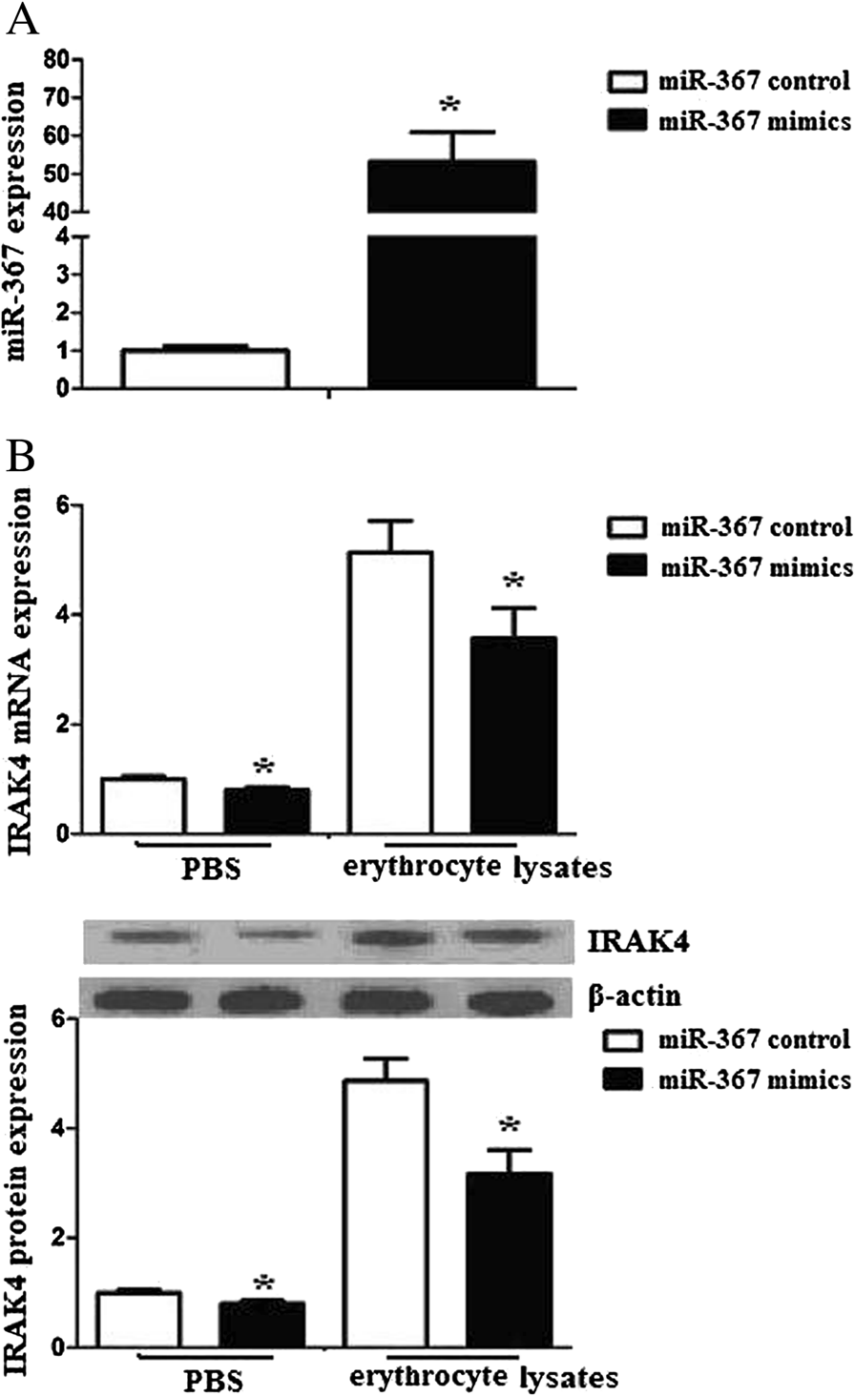
miR-367 regulates IRAK4 expression in microglia. **a** Microglia was transfected with miR-367 mimics or miR-367 control. After 24 h, cells were harvested, and miR-367 expression was evaluated by RT-PCR. **b** Microglia was transfected with miR-367 mimics or miR-367 control, and then was treated with PBS or erythrocyte lysates. After 48 h, cells were harvested, and IRAK4 mRNA and protein expression were evaluated by RT-PCR and western blots. Experiments performed in triplicate showed consistent results. Data are presented as the mean ± SD of three independent experiments. *P < 0.05

To detect the relationship of miR-367 and IRAK4, we transfected microglia with miR-367 mimics or miR-367 control. We found that transfection of miR-367 improved miR-367 mRNA expression (Fig. 2a). We further transfected microglia with miR-367 mimics or miR-367 control, and then treated the microglia with erythrocyte lysates. Microglia transfected with miR-367 had dramatically reduced IRAK4 expression (Fig. 2b), which suggests that miR-367 inhibits erythrocyte lysate-induced IRAK4 expression in microglia.

### IRAK4 is a direct target of miR-367 in microglia

**Fig. 3:**
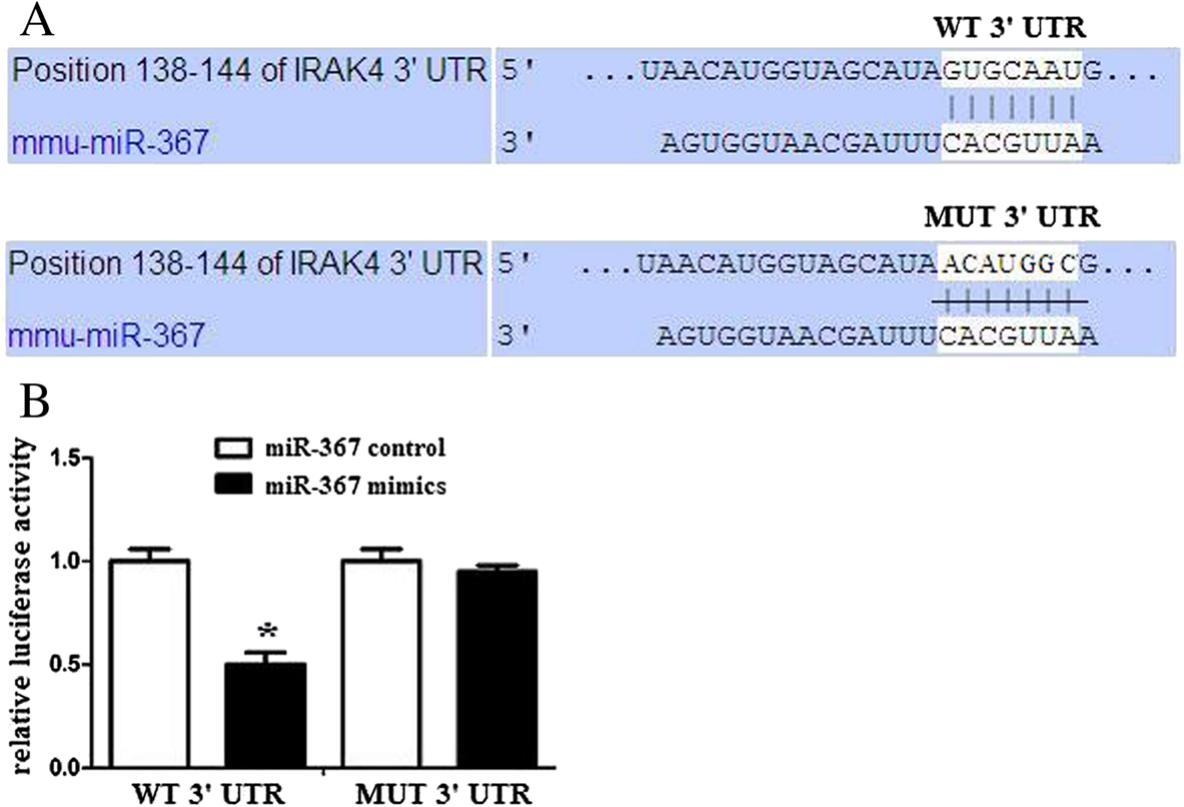
IRAK4 is a direct target of miR-367 in microglia. **a** A IRAK4 3′UTR fragment containing wild-type or mutant miR-367-binding sites was cloned downstream of the luciferase reporter gene. The region of the IRAK4 mRNA 3′UTR predicted to be targeted by miR-367 as indicated. **b** Luciferase activity assays using reporters with wild-type or mutant IRAK4 3′UTRs were performed after cotransfection with miR-367 mimics or control in microglia. The luciferase activity of the control transfection in each experiment was used to normalize the data, and the luciferase activity of the control transfection was set equal to 1. Experiments performed in triplicate showed consistent results. Data are presented as the mean ± SD of three independent experiments. *P < 0.05

We hypothesized that IRAK4 is a direct target of miR-367 in microglia. The 3′-UTR of IRAK4 mRNA contains a putative miR-367 target sequence based on the target prediction program TargetScan (www.targetscan.org) (Fig. 3a). We tested this interaction using a Dual-Luciferase reporter system. Co-expression with miR-367 mimics significantly suppressed the activity of a firefly luciferase reporter containing wild-type IRAK4 3′-UTR but had no effect on a reporter with a mutated IRAK4 3′-UTR (Fig. 3b). These results indicate that miR-367 likely suppresses IRAK4 expression by directly binding target sites in the IRAK4 3′-UTR.

### miR-367 inhibited NF-κB activation and proinflammatory mediators production

**Fig. 4:**
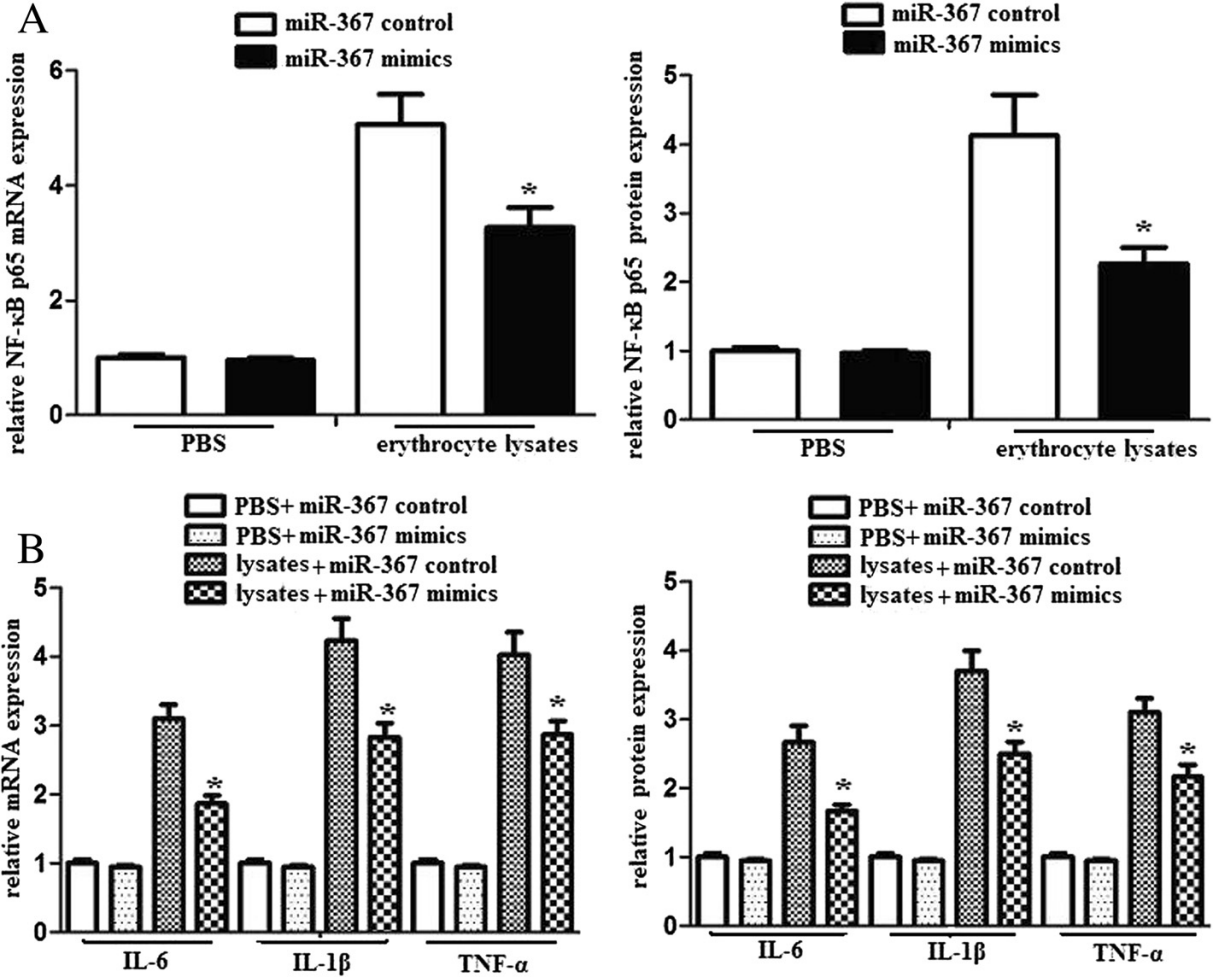
miR-367 inhibited NF-κB activation and the downstream production of proinflammatory mediators in microglia. Microglia was transfected with miR-367 mimics or miR-367 control. And then, microglia (1 × 10^5^) was stimulated with PBS or erythrocyte lysates for 48 h. **a** NF-κB p65 mRNA and protein expression were analyzed using by RT-PCR and western blots. **b** IL-6, IL-1β and TNF-α mRNA and protein expression were evaluated by RT-PCR and ELISA. Experiments performed in triplicate showed consistent results. Data are presented as the mean ± SD of three independent experiments. *P < 0.05

IRAK4 encodes a kinase that activates NF-κB in both the Toll-like receptor (TLR) and T-cell receptor (TCR) signaling pathways, which induces the production of various proinflammatory mediators. We detected the effects of miR-367 on the NF-κB pathway of erythrocyte lysate-treated microglia. The results demonstrated that upregulated miR-367 attenuated NF-κB p65 mRNA and protein levels in the nuclei of erythrocyte lysate-treated microglia (Fig. 4a). In addition, we also found that upregulated miR-367 attenuated inflammatory mediator expression of erythrocyte lysate-treated microglia (Fig. 4b).

### miR-367 attenuated inflammatory response of microglia via IRAK4

**Fig. 5:**
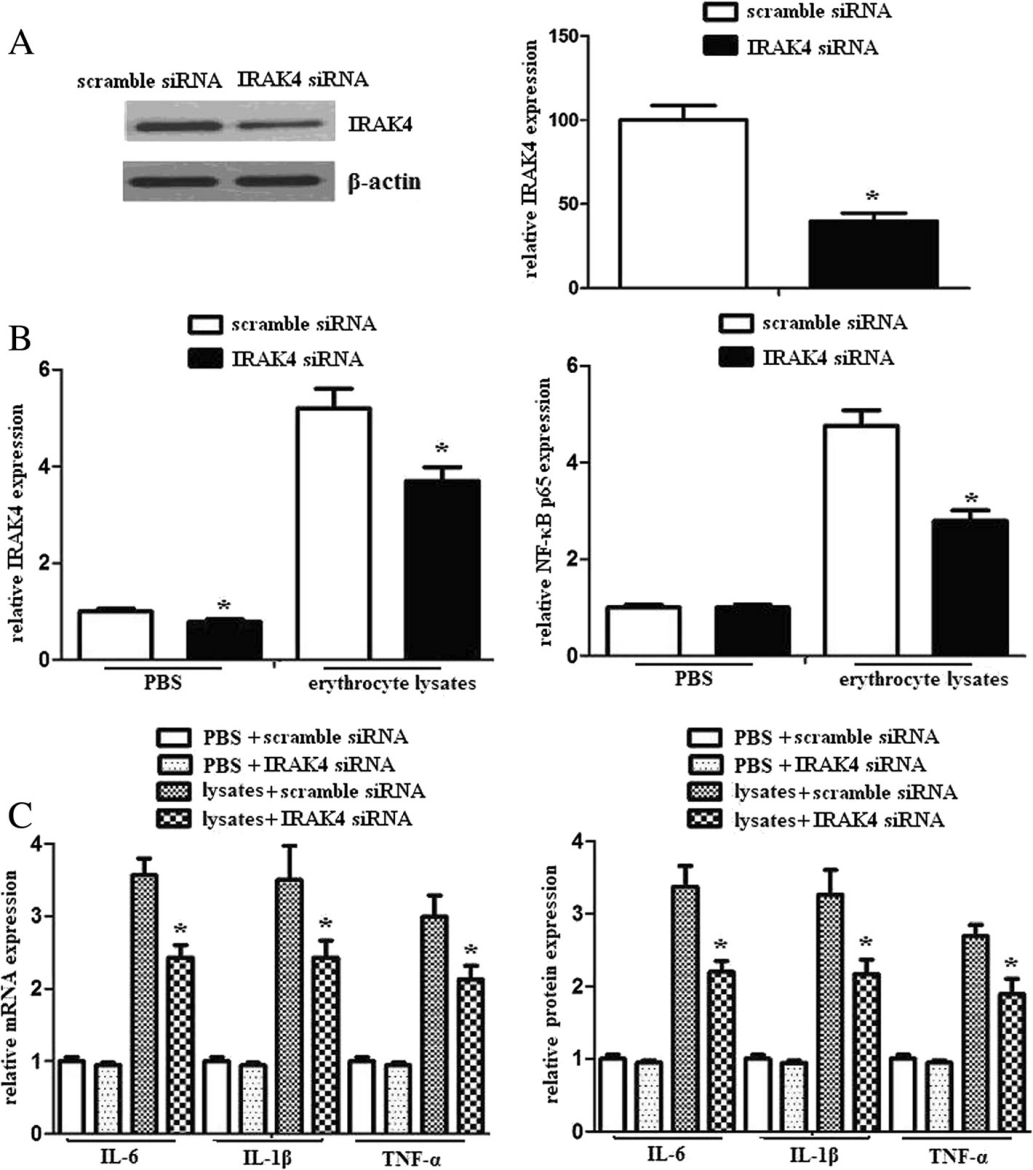
IRAK4 mediated the suppressed inflammatory response induced by miR-367 in erythrocyte lysate-treated microglia. **a** Detection of the inhibition efficiency of siRNAs against IRAK4. Microglia was transfected with scramble siRNA or IRAK4 siRNA and then exposed to erythrocyte lysates. After 48 h, IRAK4 protein expression was analyzed using western blots. **b** Microglia was transfected with scramble siRNA or IRAK4 siRNA and then exposed to PBS or erythrocyte lysates. After 48 h, IRAK4 and NF-κB p65 protein expression were analyzed using western blots. **c** Microglia was transfected with scramble siRNA or IRAK4 siRNA and then exposed to PBS or erythrocyte lysates. After 48 h, IL-6, IL-1β, and TNF-α mRNA and protein expression were evaluated by RT-PCR and ELISA. Experiments performed in triplicate showed consistent results. Data are presented as the mean ± SD of three independent experiments. *P < 0.05

To identify whether miR-367 attenuated inflammatory response of microglia via IRAK4, we utilized siRNA to inhibit IRAK4 expression and observed the inflammatory response of microglia. We found that IRAK4 siRNA clearly decreased IRAK4 protein expression. However, scramble siRNA had no similar effect (Fig. 5a, b). In addition, inhibition of IRAK4 significantly decreased NF-κB activation in the nuclei and the downstream production of proinflammatory mediators. However, scramble siRNA had no similar effect (Fig. 5b, c). These data demonstrated that miR-367 attenuated inflammatory response of microglia via IRAK4.

### miR-367 inhibits inflammation response in vivo

**Fig. 6:**
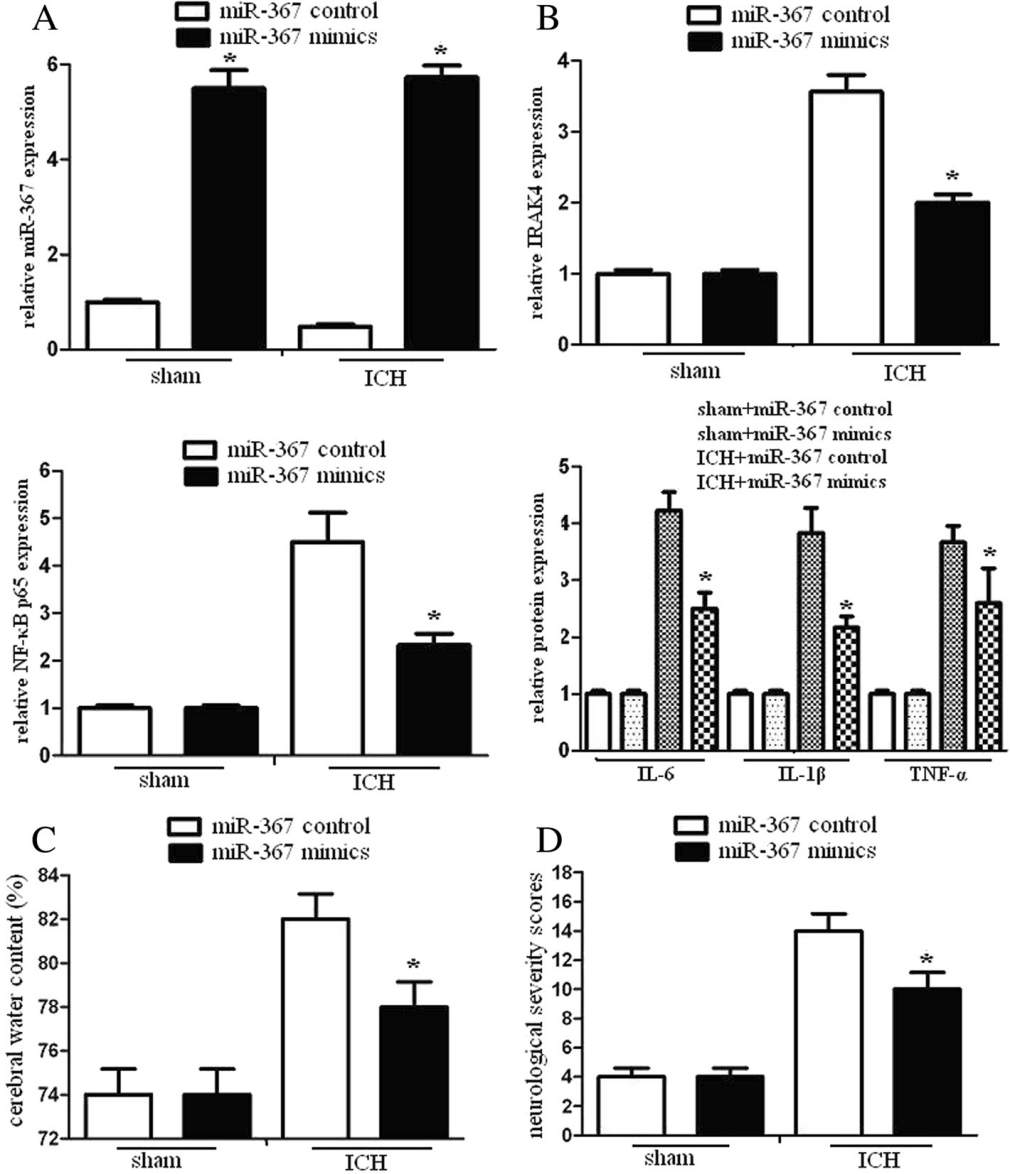
miR-367 inhibited inflammation response and improved neurological injury in vivo. Mice were received an intracerebral ventricular injection of miR-367 mimics or miR-367 control 10 min after ICH and sacrificed 48 h after ICH. The brains were dissected. **a** The mRNA levels of miR-367 were assayed by qRT-PCR assay. **b** Western blotting was used to evaluate IRAK4, NF-κB p65, IL-6, IL-1β, and TNF-α expression of brain tissues. **c** The cerebral water content of mice was analyzed. **d** The neurological deficit tests were determined by neurological severity scores, a composite of motor, sensory, reflex, and balance tests. Neurological function was graded on a scale of 1 to 18, and repeated three times. Experiments performed in triplicate showed consistent results. Data are presented as the mean ± SD of three independent experiments. *P < 0.05

To identify whether miR-367 could inhibit inflammation response in vivo, we analyzed IRAK4, NF-κB p65, and inflammatory cytokines expression in mice after ICH. We found that administration of miR-367 significantly improved the miR-367 level in vivo, and miR-367 could significantly inhibit IRAK4, NF-κB p65, IL-6, IL-1β, and TNF-α expression in brain tissues after ICH (Fig. 6a, b). These data showed that miR-367 could inhibit inflammation response in vivo.

### miR-367 reduces brain damage in ICH

In mice subjected to ICH for 48 h, water content in mice brains and neurological injury were observed. The results demonstrated that the water content in mice brains and neurological injury significantly increased compared to sham-operated animals. To determine the contribution of miR-367 to neurological function, i.c.v. injection of miR-367 control or mimics were administered 10 min after ICH. We found that administration of miR-367 significantly reduce water content and neurological injury (Fig. 6c, d). These analyses showed that miR-367 could ameliorate the neurological symptoms and improve brain function after ICH.

## Discussion

ICH is a common and devastating cerebral disease with a higher morbidity and mortality than ischemic stroke [22–24]. ICH-induced brain injury involves multiple mechanisms. The primary injury is due to the disruption of adjacent tissue and mass effect. The secondary brain damage occurs as a result of hematoma, inflammation, local release of ROS, and perihematomal edema [25–27].

miRNAs are a recently discovered class of small, noncoding RNAs with gene-regulating functions. It has been shown that miRNAs regulate the expression of protein-coding genes at the post-transcriptional level through imperfect base pairing with the 3′-UTRs of their target mRNAs [28–30]. Much evidence suggests that miRNA dysregulation may contribute to many types of human disease, including neuronal disorders [31–33]. However, the miRNA expression profiles of microglia after ICH are very limited. Although microglial activation is considered to be a hallmark of neuroinflammation, whether the expression of proinflammatory cytokines is regulated by miRNAs during microglial activation is still known.

Previous studies showed that the recognition of TLRs to pathogen-associated molecular patterns (PAMPs) could initiate protective immune responses. Studies of TLR signaling have also demonstrated that ligation of TLRs induced the recruitment and activation of IRAK4 [34–36]. Upon binding of a TLR/MyD88 complex, IRAK4 can act as a kinase that functions upstream of and phosphorylates IRAK. Then, the phosphorylated IRAK mediates the recruitment of TRAF6 to the receptor complex, resulting in transcription of pro-inflammatory cytokines [37–39].

Previously, we found that the expression level of miR-367 was significantly downregulated in microglia treated with erythrocyte lysates. Related studies showed that miR-367 promoted pancreatic cancer invasion in vitro and metastasis in vivo through downregulating Smad7 [40]. The results suggest that miR-367 may be a promising therapeutic target for the treatment of human pancreatic cancer. In addition, increasing evidence suggests that several microRNAs (miRNAs) are involved in early embryonic development and embryonic stem cell formation, known as embryonic stem cell (ESC)-specific miRNAs. The development of this miR-302/367-mediated iPSC (termed mirPSC) may provide tools to deal with the obstacles facing some current iPSC reprogramming methods [41].

In this study, we identified an inverse relationship between miR-367 and IRAK4 expression. The 3′UTR of IRAK4 mRNA contained conserved miR-367 binding sites, and we showed that miR-367 directly regulated IRAK4 expression through these 3′UTR sites. Our results indicate that miR-367 downregulation leads to increased IRAK4 expression of microglia after ICH.

We further detected the effects of miR-367 on the NF-κB pathway of erythrocyte lysate-treated microglia. We found that upregulated miR-367 attenuated NF-κB and inflammatory mediator expression of erythrocyte lysate-treated microglia. In addition, we demonstrated that inhibition of IRAK4 significantly decreased NF-κB activation and the downstream production of proinflammatory mediators. These data demonstrated that miR-367 attenuated inflammatory response of microglia via IRAK4.

Lastly, we found that over-expression of miR-367 significantly inhibited IL-6, IL-1β, and TNF-α expression in brain tissues, reduced water content and neurological injury after ICH. These data showed that miR-367 could inhibit inflammation response in vivo, meliorate the neurological symptoms, and improve brain function after ICH.

## Conclusion

In conclusion, we identified a strong correlation between miR-367 and IRAK4, and the effect of miR-367 on microglial activation after ICH. Therefore, the miR-367 represents a potential therapeutic target for ICH.
